# A Novel Balanced-Lethal Host-Vector System Based on *glmS*

**DOI:** 10.1371/journal.pone.0060511

**Published:** 2013-03-28

**Authors:** Kwangsoo Kim, Jae Ho Jeong, Daejin Lim, Yeongjin Hong, Misun Yun, Jung-Joon Min, Sahng-June Kwak, Hyon E. Choy

**Affiliations:** 1 Department of Microbiology, Chonnam National University Medical School, Dong-gu, Gwangju, Republic of Korea; 2 Department of Nuclear Medicine, Chonnam National University Medical School, Dong-gu, Gwangju, Republic of Korea; 3 Department of Biochemistry, Dankook University Medical College, Chungnam, Cheonan, Anseo, Korea; Mie University Graduate School of Medicine, Japan

## Abstract

During the last decade, an increasing number of papers have described the use of various genera of bacteria, including *E. coli* and *S. typhimurium*, in the treatment of cancer. This is primarily due to the facts that not only are these bacteria capable of accumulating in the tumor mass, but they can also be engineered to deliver specific therapeutic proteins directly to the tumor site. However, a major obstacle exists in that bacteria because the plasmid carrying the therapeutic gene is not needed for bacterial survival, these plasmids are often lost from the bacteria. Here, we report the development of a balanced-lethal host-vector system based on deletion of the *glmS* gene in *E. coli* and *S. typhimurium*. This system takes advantage of the phenotype of the GlmS^−^ mutant, which undergoes lysis in animal systems that lack the nutrients required for proliferation of the mutant bacteria, D-glucosamine (GlcN) or N-acetyl-D-glucosamine (GlcNAc), components necessary for peptidoglycan synthesis. We demonstrate that plasmids carrying a *glmS* gene (*GlmS^+^p*) complemented the phenotype of the GlmS^−^ mutant, and that *GlmS^+^*p was maintained faithfully both *in vitro* and in an animal system in the absence of selection pressure. This was further verified by bioluminescent signals from *GlmS* ^+^pLux carried in bacteria that accumulated in grafted tumor tissue in a mouse model. The signal was up to several hundred-fold stronger than that from the control plasmid, pLux, due to faithful maintenance of the plasmid. We believe this system will allow to package a therapeutic gene onto an expression plasmid for bacterial delivery to the tumor site without subsequent loss of plasmid expression as well as to quantify bioluminescent bacteria using *in vivo* imaging by providing a direct correlation between photon flux and bacterial number.

## Introduction

Cancer therapy using bacteria takes advantage of multiple types of bacteria, including *Salmonella*, *Escherichia*, *Clostridium*, *Proteus* and *Streptococcus*, due to their tendency to accumulate preferentially in tumor tissue, although the mechanism that mediates this remains unknown [1]. Another obvious advantage of the use of bacteria as antitumor agents is that they can be engineered to deliver a specific protein of interest directly to the tumor mass. This feature makes bacterial therapy attractive compared to other therapies, including chemotherapy and radiation therapies, which are often toxic to normal cells.

Our laboratory has been developing bacterial therapy using *E. coli* and the non-virulent strain *Salmonella typhimurium*, which is defective in ppGpp synthesis, [2], [3] to deliver therapeutic/bio-imaging proteins specifically to tumor masses in murine models [4]–[6]. In general, the expression of cargo proteins in these bacterial strains is achieved by introducing recombinant plasmids carrying genes encoding the desired proteins. Therefore, plasmid stability is the most critical parameter for the successful delivery of cargo proteins into the tumor mass. However, the use of antibiotic resistance genes as a selective determinant for plasmid maintenance is impractical *in vivo*. This problem was first addressed by the construction of a balanced-lethal system in which the *asd* gene of *St. mutans* was introduced in a plasmid that complements an *asd* mutation in the chromosome of the *Salmonella* strain [7]. The *asd* gene encodes aspartate-semialdehyde dehydrogenase, an enzyme required for the synthesis of diaminopimeic acid (DAP), an essential component of the cell wall peptidoglycan of Gram-negative bacteria [8]. In the absence of DAP, *asd* mutants quickly undergo lysis. Since DAP is not present in mammalian tissues, this balanced-lethal system ensures that all surviving *asd* mutant *Salmonellae* carry the recombinant Asd+ plasmid [7].

In this study, we describe the development of a balanced-lethal host system based on an enzyme essential for peptidoglycan synthesis in *E. coli* and *S. typhimurium*. The amino sugars D-glucosamine (GlcN) and N-acetyl-D-glucosamine (GlcNAc) are essential components of the peptidoglycans of bacterial cell walls and the lipopolysaccharides of the outer membrane in Gram-negative bacteria. Genes for both the uptake [9] and metabolism [10], [11] of these amino sugars are encoded by five genes of the *nag* regulon [12], [13] [14]. In the absence of these amino sugars, bacteria must synthesize glucosamine-6-phosphate (GlnC-6-P) from fructose-6- phosphate and glutamine via the enzyme glucosamine-6-phosphate synthase (L-glutamine: D-fructose-6-phosphateamidotransferase; EC 2.6.1.16), which is encoded by the gene *glmS*. GlcN-6-P is then converted to GlcN-1-P by GlmM and subsequently acetylated to yield UDP-GlcNAc by GlmU. UDP-GlcNAc is the primary cytoplasmic intermediate in the synthesis of lipid A and peptidoglycan [15]. Mutant bacteria defective in the synthesis of these intermediates are strictly dependent on the presence of exogenous GlcN or GlcNAc. Elimination of these compounds from culture conditions causes a rapid loss of viability and cell lysis [16]–[18]. Thus, we constructed a balanced-lethal system in which the *glmS* gene is present in a plasmid that complements *glmS* mutation in the chromosome of the *E. coli* and *S.typhimurium*. We demonstrate that GlmS^−^ mutant bacteria failed to accumulate in tumor tissue in a murine model and that GlmS^−^ mutant bacteria carrying the GlmS^+^ plasmid proliferated as well as wild-type bacteria. We also show that only 1/1000∼1/10,000 wild-type bacteria maintained the GlmS^+^ plasmid, while all GlmS^−^ mutant bacteria retained the GlmS^+^ plasmid, thus confirming its function in a balanced-lethal host system.

## Materials and Methods

### Bacterial strains and constructions

The bacterial strains used in this study are summarized in Table 1. All *E. coli* strains were derived from the MG1655 background. The GlmS^−^ mutant strain (IBPC750) was kindly provided by J. Plumbridge (France). The *glmS*: *tet^R^* was transferred to test strains by P1 phage [19]. The streptomycin resistant strain (CH1436) was obtained from colonies grown on LB plates containing streptomycin (10 µg/ml). The GlmS^−^ mutant *S. typhimurium* (SKS1001) was constructed from SCH2005 (14028s) by the method developed by Datsenko and Wanner [20]. A *DNA* fragment carrying a *kan^R^* cassette in place of the *glmS* open reading frame was generated by PCR amplification using the primer pairs pKD *^S.t^glmS* 5′ (TTA CTC AAC CGT AAC CGA TTT TGC CAG GTT ACG CGG CTG GTC AAC GTC GGT GCC TTG ATT GTG TAG GCT GGA GCT GCT TCG AA) and pKD *^S.t^glmS* 3′ (ATG TGT GGA ATT GTT GGC GCG ATC GCG CTT CGT GAT GTA GCT GAA TCC TTC TTG AAG GTC ATA TGA ATA TCC TCC TTC GTT CC). The ΔppGpp/GlmS^−^ mutant *Salmonella* (SKS1002) was constructed by transduction using p22 phage.

**Table 1:** Bacterial strains and plasmids used in this study.

Strains	Description	Reference or source
***E. coli***		
MG1655	Wild type (*E.coli* K-12)	ATCC
CH1018	*Δarg/lac*	Kim, et al. [36]
HJ1020	*Δasd*	Gallan, et al.[7]
IBPC750	*GlmS::tet*	J.Plumbridge **(**FR)
CH1436	Streptomycin resistant CH1018	This work
CKS1001	CH1436, *glmS:Tet^r^*	This work
*^E.c^GlmS^+^p*	pUC19 containing *^E.c^GlmS* (of *E. coli*)	This work
*pGFP*	pUC19 containing GFP	Clontech
*^E.c^GlmS^+^pGFP*	*^E.c^GlmS^+^p* containing GFP	This work
*pLux*	pUC19 containing Lac*P*::LuxCDABE	This work
*^E.c^GlmS^+^pLux*	*^E.c^GlmS^+^p* containing Lac*P*::LuxCDABE	This work
***S.typhimurium***		
SCH2005	*S.Typhimurium* 14028s	ATCC
SHJ2037	SCH2005, *relA::kan, spoT::cat, Kan^r^, Cam^r^*	Song, et al. [31]
SMR2130	SHJ2037, *ΔrelA,ΔspoT*	This work
SKS1001	SCH2005, *glmS::kan, Kan^r^*	This work
SKS1002	SMR2130, *glmS::kan, Kan^r^*	This work
*^S.t^GlmS^+^p*	pBAD24 containing *^S.t^GlmS (*of *S. typhimurium*)	This work
*^S.t^GlmS^+^pLux*	*^S.t^GlmS^+^p* containing Lac*P*::LuxCDABE	This work

### Plasmids

The luminescence-expressing plasmid (*pLux*) was previously decribed [5]. Briefly, *pLux* containing the *lux* operon (*luxCDABE*) of *Photobacterium leiognathi* was inserted into the pUC19 plasmid backbone using an *Xba*I restriction enzyme site [21].The original *pLux* contains 9.5 kb of the *lux* operon and approximately 700 bp of unknown sequence upstream. The unknown sequence was replaced by the *lac* promoter sequence as follows. The *lux* operon without the upstream sequence was PCR-amplified using two primers: lux1 (5′-GGGAATTCTATACCGAAACTACATAC-3′) and lux2 (5′-GGGTCTAGAGCACTTAATGCCGCTACT-3′). This 8.8 kb DNA fragment was digested with *Xba*I and *Eco*RI and ligated into the same site in pUC19, generating the construct pΔNlux. The *E. coli lac* promoter sequence was obtained using the forward primer 5′-GGGAATTCCATGGTCATAGCTGTTTC-3′ and the reverse primer 5′-GTGAGCTCGGGATCCTCTAGAGTCGA-3′. This 100 bp fragment was cloned into the pGEM-T Easy vector (Promega, Madison, WI, USA), digested with *Eco*RI, and ligated into the same site in the *pΔNlux* vector, generating pLac*P*::Lux (*pLux*).

The expression vector for *glmS* was constructed as follows. The *glmS* gene was amplified from *E. coli* genomic DNA using the forward primer 5′-GGAAGCTTATGTGTGGAATTGTTGGCG-3′ and the reverse primer 5′-GGTCTAGATTACTCAACCGTAACAGATTTTG-3′. This 1.8 kb fragment was digested with HindIII and XbaI and ligated into the same site in the pUC19 vector, generating *^E.c^GlmS^+^p*.

To construct a plasmid containing both the *lux operon* cassette and *glmS*, the *glmS* gene from *E. coli* (MG1655) was amplified by PCR using the forward primer 5′- AAGTCGACATGTGTGGAATTGTTGGCG-3′ and the reverse primer 5′- GGGTCGACTTACTCAACCGTAACAGATTTTG-3′.This 1.8 kb fragment was digested with *Sal I* and ligated into the same site in the *pLux* vector, generating *^E.c^GlmS^+^pLux*.

To construct *^E.c^GlmS^+^pGFP*, the GFP gene was amplified by PCR using the *pEGFP* plasmid as a template. The primers used were 5′- GGCCCGGGGTGAGTTAGCTCACTCATTAG (forward) and 5′- AAACCCGGGGAA TTCTAGAGTCGCCGC (reverse). This 1.1 kb fragment was digested with *SmaI* and ligated into the same site in the *^E.c^GlmS^+^p* vector, generating *^E.c^GlmS^+^pGFP*.

The *glmS* gene of *S. typhimurium* (*^S.t^glmS*) was amplified with the forward primer 5′- GGGCTAGAATGTGTGGAATTGTTGGC -3′ and the reverse primer 5′- GGGAAGCTTTTACTCTACGGTAACCGATTTC -3′ using *S. typhimurium* (*14028s*) genomic DNA as a template. Using XbaI and HindIII restriction sites, the PCR product was inserted into the pBAD24 vector, resulting in *^Sc^GlmS^+^p*.

To construct a plasmid containing both the *lux* cassette and *^S.t^glmS^+^ p*, the *glmS* gene from the genomic DNA of 14028s was amplified by PCR. This 1.8 kb fragment was digested with *SalI* and ligated into the same site in the *pLux* vector, generating *^S.t^glmS^+^ pLux*.

### Bacterial growth conditions

Bacteria were grown in Luria–Bertani (LB) medium (Difco Laboratories, USA) containing 1% NaCl or M9 minimal medium supplemented with 0.2% glucose, 1 μg/ml thiamine, and 1 μg/ml calcium pantothenate. For solid support medium, 1.5%-bacto agar was included. All media were supplemented with antibiotics as follows: ampicillin at 100 µg/ml, tetracycline 15 µg/ml and streptomycin 10 µg/ml. When needed, N-acetyl-D-glucosamine (GlcNAc) was added (100 µg/ml).

### Cell culture

HeLa, 4T-1, and CT26 cell lines were grown in high-glucose Dulbecco's modified Eagle medium (DMEM), and ASPC1 in RPMI1640 medium, both containing 10% FBS and 1% penicillin-streptomycin.

### Isolation and culture of peritoneal macrophages

Peritoneal macrophages were isolated from BALB/c and BALB/c athymic nu^−^/nu^−^ mice (20–30 g body weight) purchased from the Samtako Company, Korea. Peritoneal macrophages were harvested from mice 3 days after intraperitoneal injection of thioglycollate (2 ml, 4%) and plated in DMEM containing 10% FBS. After 4 hours incubation at 37°C, non-adherent cells were removed and adherent cells were used for experiments.

### Salmonella invasion assay

Bacterial invasion assays were performed as described previously. Overnight cultures of *S. typhimurium* (14028S) were grown at 37°C in Luria-broth (LB) medium. *S. typhimurium* was inoculated into fresh cultures and grown for 4 hrs at 37°C, and then resuspended at the appropriate dilution in cell culture medium for infection of cell monolayers at an MOI 1∶10 for 30 min. Cells (1×10^5^) were seeded in 24-well plates and grown in DMEM with 10% FBS at 37°C in a 5% CO_2_ incubator. Infected cells were washed three times with PBS (pH 7.4). DMEM containing gentamicin (10 µg/ml; Sigma Chemical) was added, and the mixtures were incubated for 30 min. Intracellular bacteria were harvested by extraction with lysis buffer (0.05% Triton X-100 in PBS, pH 7.4) in triplicate for colony counting on brain–heart infusion agar plates supplemented with GlcNAc.

### Protein preparation and Western blot analysis

Protein samples were boiled for 5 min and separated by SDS-PAGE. The separated proteins were then transferred electrophoretically to a nitrocellulose membrane. The membrane was blocked with 5% skim milk and probed with a mouse anti-GFP antibody (1∶5000; Sigma-Aldrich, UK) at 4°C overnight. The membrane was then incubated with anti- mouse IgG antibody linked to horseradish peroxidase (Sigma-Aldrich, UK) for 1 hr and bound proteins were visualized by ECL (Amersham Biosciences).

The bacterial spent media and bacterial pellets were prepared as follows: the pellets were lysed by sonication in phosphate-buffer saline with lysis buffer (10 mM lysozyme, 10% SDS). The spent media were filtered (0.22 µm pore filter) and the proteins were precipitated with 10% trichloroacetic acid (1 hr, 4°C).

### Measurement of plasmid stability

Overnight cultures were subcultured in fresh media (1/1000) without ampicillin every 12 hours for 4 days. Samples were taken every 24 hrs and serially diluted in sterile 0.9% NaCl, and appropriate volumes were spread in triplicate on LB agar plate containing GlcNAc with or without ampicillin. The number of colonies was used to calculate the concentration of total viable cells and the percentage of plasmid-carrying bacteria.

### β-galactosidase assay

β-galactosidase assay was performed as described by Miller [22] using bacterial pellets that were permeabilized with Koch's lysis solution [23] or filtered spent media. Formation of ONP was determined as A_420_/min/ml.

### Animal model

Five- to six-week-old male BALB/c (for 4T-1 and CT26) and BALB/c athymic nu-/nu- mice (for ASPC1) (20–30 g body weight) were purchased from the Samtako Company, Korea. All animal care, experiments, and euthanasia were performed in accordance with protocols approved by the Chonnam National University Animal Research Committee. Animals were anesthetized with isoflurane (2%) during imaging or a mixture of ketamine (200 mg kg^−1^) and xylasine (10 mg kg^−1^) for surgery. Mice carrying subcutaneous tumors were generated as follows: *in vitro* cultured tumor cells were harvested, suspended in 100 µl PBS and injected subcutaneously into the right thigh of each mouse: 1×10^6^ cells for 4T-1 and CT26, and 1×10^7^ cells for ASPC1. Tumor volumes (mm^3^) were estimated using the formula (L × H × W)/2 where L is the length, W is the width, and H is the height of the tumor in millimeters [21].

### Injection of bacteria into animals

Bioluminescent *E. coli* or *S. typhimurium* (1×10^8^) suspended in 100 µl PBS were injected intravenously into tumor-bearing mice through the lateral tail vein using a l cc insulin syringe [21].

### Optical bioluminescence imaging

To image bacterial bioluminescence, anesthetized animals were placed in the light-tight chamber of the IVIS100 (Caliper, Hopkinton, MA, USA) equipped with a cooled charged couple detector (CCD) camera. Photons emitted from luciferase-expressing bacteria were collected and integrated over one-minute periods. Pseudocolor images representing photon counts were overlaid on photographs of the mice using Living Image software v. 2.25 (Caliper, Hopkinton, MA). A region of interest (ROI) was selected manually based on signal intensity. The area of the ROI was kept constant, and the intensity was recorded as the maximum number of photons (photons s^−1^ cm^−2^ sr^−1^) within a ROI [21].

### Preparation of tissue extracts

Spleen and liver extracts of mice were prepared by adding organ homogenates directly to M9 media. The bone marrow extract was prepared from cancellous bone and marrow cavity of pig femur. The tissue sample was lysed using French pressure (1,000 *psi*) and homogenizer, and the extract was taken after centrifugation (5,000 rpm, 5 min, Eppendorf) and filtration (0.45 µm).

### Statistical analysis

Statistical analysis was performed using the SPSS 18.0 statistical package (SPSS Inc., Chicago, IL, USA). A two-tailed Student's *t-*test was used to determine the statistical significance of tumor growth between the control and treatment groups. A *P* value of <0.05 was considered statistically significant.

## Results

### Characterization of E. coli GlmS^+^p vector in vitro

**Figure 1:**
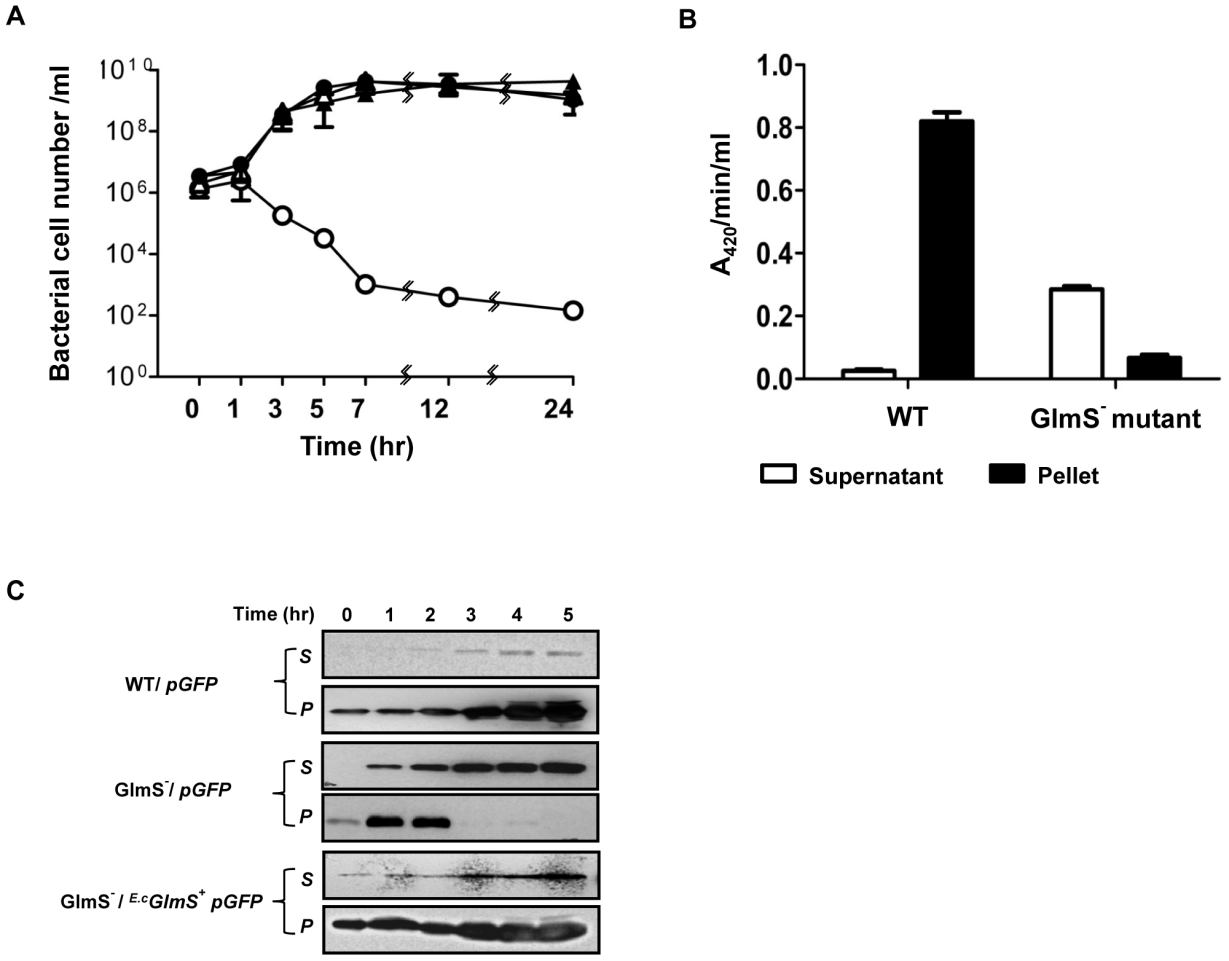
Phenotype of GlmS^−^ mutant *E. coli*. (A) Growth of GlmS^−^ mutant *E. coli* under various media conditions. GlmS^−^ mutant *E. coli* (CKS1001, open circles) and GlmS^−^ mutant *E. coli* carrying *^E.c^GlmS^+^p* (closed circles) grown overnight in LB supplemented with 0.2% GlcNAc were diluted 50-fold in minimal media or media supplemented with 0.2% GlcNAc (open triangles) and grown for 24 hrs. Wild-type parental *E. coli* (CH1436, closed triangles) were grown in the same way in minimal media. Samples were taken at the indicated times for CFU determination on supplemented LB plates. (B) GlmS^−^ mutant (CKS1001) or parental wild-type *E. coli* (CH1436) carrying ϕ*hdeAB*p:*lacZYA* grown overnight in LB supplemented with 0.2% GlcNAc were diluted 50-fold in minimal media and grown for 5 hrs. β-galactosidase activity (A_420_/min/ml) in the supernatants and lysed pellets was determined. (C) GlmS^−^ mutant bacteria (CKS1001) carrying *^E.c^GlmS^+^pGFP* or *pGFP* grown overnight in LB supplemented with 0.2% GlcNAc were diluted 50-fold in minimal media and cultured for the indicated times. Wild-type parental *E. coli* (CH1436) carrying *pGFP* was grown the same way in minimal media. Samples were taken at the indicated times, the supernatant (*s*) and pellet (*p*) fractions were isolated, and the fractions were separated by 12% SDS-PAGE for determination of GFP by Western blotting.

GlmS^−^ *E. coli* requires exogenous GlcN/GlcNAc for its survival [24]. GlmS^−^ mutant bacteria was generated and its phenotype was assessed by culturing the mutant strain (CKS1001) in minimal media (M9) (Fig. 1A). Bacteria grown in the presence of supplemental GlcNAc were subcultured into minimal media and samples taken at the indicated times were plated on LB agar plates supplemented with GlcNAc for viable cell counting (Colony Forming Units, CFU). On LB plates, GlmS^−^ mutant bacteria replicated for a few rounds and then underwent lysis, indicating that an insufficient amount of nutrients for survival of GlmS^−^ mutants was present in LB (see below). The assay revealed that the viability of the GlmS^−^ mutant reduced drastically from 10^6^ CFU to 10^2^ CFU over 24 hrs. When GlcNAc was present in the culture media, however, the CFUs of the mutant increased in size, as did those of the wild type. These phenotypes were similar to those previously observed in GlmM^−^ mutant bacteria [25]. Subsequently, the fate of GlmS^−^ mutant bacteria in the absence of supplemental GlcNAc was further examined by determining whether or not bacterial death was due to cell lysis. A GlmS^−^ mutant carrying a plasmid, in which DNA fragment of *hdeABp::lacZYA* [26] was cloned, was used to determine the degree of bacterial lysis in the absence of GlcNAc (Fig. 1B). Samples were taken 5 hr after subculturing in minimal media and used for β-galactosidase assay of the supernatant (without any additional processing) and in the bacterial pellets (after treatment with cell lysis solution) [23]. A_420_/min/ml (product formation) instead of A_420_/min/ml/A_600_ (specific enzyme activity) was determined, since the A_600_ value (cell mass) of the GlmS^−^ mutant would be meaningless due to the significant decrease in CFU (over 10^4^-fold) over the period of 5 hrs. The sum of A_420_/min/ml in the supernatant and pellet was 0.85 for wild-type bacteria and 0.35 for GlmS^−^ mutant bacteria. However, the A_420_/min/ml determined for the GlmS^−^ mutant was predominantly in the supernatant (>80%), while that for the wild type was exclusively in the pellet, suggesting that GlmS^−^ mutant bacteria undergo lysis under these culture conditions. To further verify lysis of the GlmS^−^ mutant in the absence of supplemental GlcNAc, wild type and GlmS^−^ mutant *E. coli* were transformed with *pGFP* and cultured in minimal media as in Figure 1A. Supernatant and pellet samples were taken at the indicated times and analyzed by Western blot analysis for GFP using a GFP-specific antibody (Fig. 1C). Samples from the culture of GlmS^−^ mutant bacteria (CKS1001) carrying *pGFP* contained GFP in the supernatant beginning at 1 hr and increasing thereafter, whereas GFP in the pellet was detected only up to the 2 hr time point. Samples from the culture of wild type bacteria (CH1018) carrying *pGFP* showed GFP exclusively in the pellet for the duration of the experiment.

**Figure 2:**
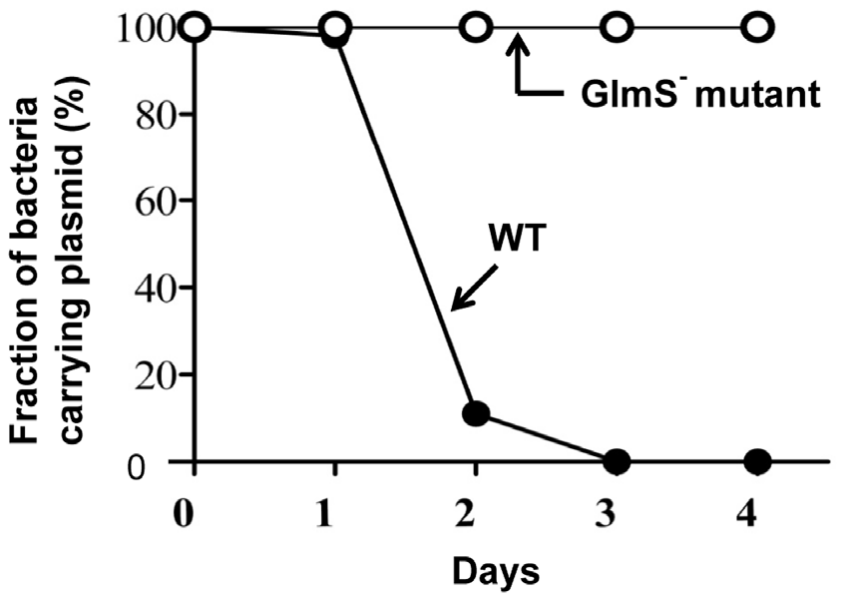
Plasmid maintenance in *E. coli* using the *glmS* system. GlmS^−^ mutant bacteria (CKS1001) or the parental strain (CH1436) carrying *^E.c^GlmS^+^p* were subcultured (1/1000) in minimal media every 12 hrs. The fraction of bacteria carrying *^E.c^GlmS^+^p* at the indicated time was determined on GlcNAc-supplemented LB plates containing ampicillin (50 mg/ml).

Based on the above observations, a balanced-lethal host-vector system was constructed in which the *glmS* gene was incorporated into a plasmid that would complement the chromosomal *glmS* mutation. A 1.8 Kb DNA fragment carrying the *glmS* of *E. coli* was obtained by PCR amplification and placed under the control of the *lac* promoter in a pUC19 plasmid (see Materials and Methods). The *GlmS^+^* plasmid (*^E.c^GlmS^+^p*) was used to transform GlmS^−^ mutant bacteria, which were then tested for complementation of the GlmS^−^ mutant phenotype (Fig. 1A). GlmS^−^ mutant bacteria carrying *^E.c^GlmS^+^p* were cultured in minimal media and assessed for CFU at the indicated times. The mutant carrying *^E.c^GlmS^+^p* survived as well as wild-type bacteria in the absence of supplemental GlcNAc. We also examined the complementation of GlmS^−^ mutant by *^E.c^GlmS^+^pGFP* (Fig. 1C). Samples from the culture of GlmS^−^ mutant bacteria carrying *^E.c^GlmS^+^pGFP* was analyzed and shown that GFP exclusively in the pellet, similar to wild-type bacteria carrying *pGFP*. Subsequently, wild-type and GlmS^−^ mutant bacteria were transformed with *^E.c^GlmS^+^p*, and maintenance of the plasmid in the absence of antibiotics was determined (Fig. 2). Bacteria were grown in minimal media and subcultured (1∶1000) every 12 hrs. Samples were taken on the indicated days to assess plasmid maintenance by plating the bacteria on plates containing ampicillin. In the wild-type background, the plasmid was lost rapidly; 92% by day 2 and over 99% by day 3. In the GlmS^−^ mutant background, no loss of the plasmid was observed for the duration of the experiment (4 days). Clearly, this result demonstrated the feasibility of using *glmS* mutant bacteria in a balanced-lethal system to maintain plasmid expression in the absence of antibiotics.

### Characterization of the E. coli GlmS^+^ p vector in a mouse model

**Figure 3:**
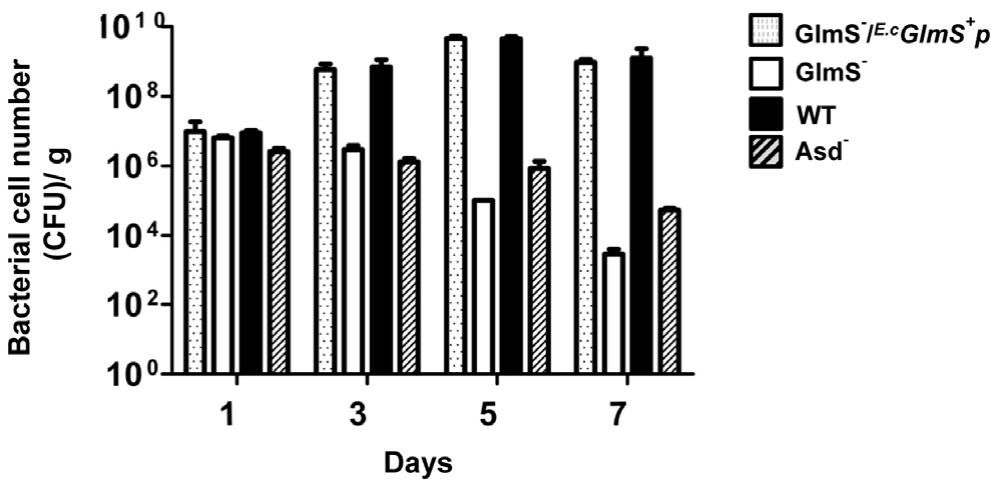
Targeting and proliferation of various mutant *E. coli* strains in CT26 tumor-bearing mice. GlmS^−^ mutant bacteria (CKS1001), GlmS^−^ mutant bacteria carrying *^E.c^GlmS^+^ p*, Asd^−^ mutant bacteria (HJ1019), and the parental wild-type *E. coli* (CH1436) were injected into CT26 tumor-bearing mice through the tail vein (1×10^8^ CFU), and the number of bacteria in the tumor tissues were counted at the indicated days by determining the number of CFU.

A prerequisite for the use of a balanced-lethal system based on *glmS* would be the absence of a sufficient supply of the nutrients required for the proliferation of GlmS^−^ mutant bacteria in an animal system. Since it has been shown that many bacterial species, including *E. coli*, are capable of targeting [4] and proliferating in tumor tissue [1], we assessed the survival capacity of GlmS^−^ bacteria in tumor tissues [21]. A mouse tumor model was created by implanting CT26 mouse colon cancer cells in the right thigh of BALB/c mice. After 14 days, wild-type or GlmS^−^ mutant bacteria (1×10^8^ CFU) with or without *^E.c^GlmS^+^p* were injected intravenously into each mouse via the tail vein. For enumeration of the bacteria in the mouse tumor model, a streptomycin-resistant mutant was generated and the allele was moved to the test strains. This was necessary to correct for contamination or bacteria pre-existing in the mice. Chromosomally-acquired streptomycin resistance is mainly due to mutations in the gene encoding the ribosomal protein S12, *rpsL* [27]. The tumor tissues were sampled on the indicated days, homogenized and spread on LB plates supplemented with GlcNAc and containing streptomycin (Fig. 3). At day 1, approximately equal numbers of bacteria were observed for both wild-type and GlmS^−^ mutant bacteria (∼1×10^6^). While the number of wild-type bacteria increased to approximately 10^9^ CFU by day 5, the number of GlmS^−^ mutant bacteria decreased to approximately 5×10^3^ CFU by day 7. In this study, Asd^−^ mutant bacteria were also enumerated. The number of Asd^−^ mutant bacteria at day 1 was similar to that of wild-type bacteria, but this value decreased to approximately 5×10^4^ CFU by day 7. Taken together, these findings demonstrate that animal systems lack sufficient amounts of the nutrients required for the proliferation of GlmS^−^ mutant bacteria, similar to Asd^−^ mutant bacteria. GlmS^−^ mutant bacteria carrying *^E.c^GlmS^+^p* proliferated as well as wild-type bacteria, demonstrating that the *glmS* gene on the plasmid was able to complement the chromosomal mutation in an *in vivo* mouse model.

**Figure 4:**
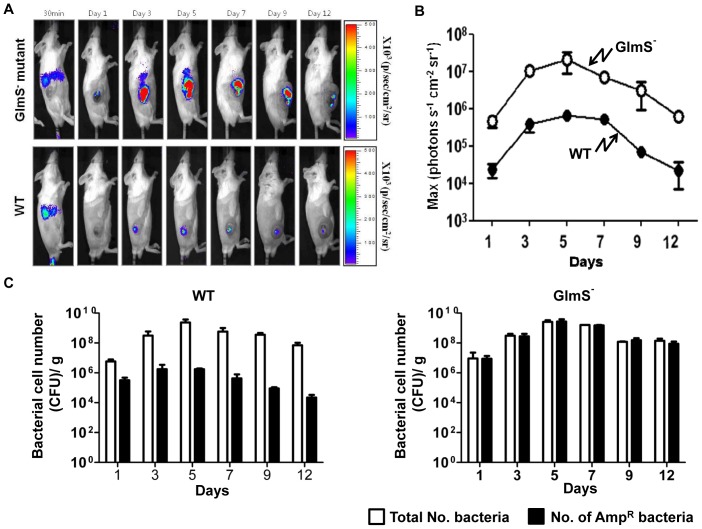
Maintenance of *pLux* in *E. coli* proliferating in tumor tissue. (A) GlmS^−^ mutant bacteria (CKS1001) and parental wild type *E. coli* (CH1436) carrying *^E.c^GlmS^+^ pLux* were injected into CT26 tumor-bearing mice through the tail vein (1×10^8^ CFU). Bioluminescent signals from *pLux* were monitored at the indicated times using an *in vivo* imaging system. (B) The photon intensity of the tumor region was plotted as a function of time for GlmS^−^ mutant and wild-type *E. coli*. The region of interest (ROI) was selected manually over the tumor region and the area was kept constant. Photon intensity was recorded as the maximum intensity (photons s^−1^ cm^−2^ sr^−1^) within the ROI. Data represent the means and SEM of three independent experiments. (C) Tumor tissues were sampled on the indicated days. The total number of bacteria and the number of bacteria carrying *^E.c^GlmS^+^ pLux* was assessed by CFU determination.

Our laboratory previously reported a quantitative and noninvasive imaging technique that enables the monitoring of bacterial migration in living subjects [5]. In this technique, bioluminescent bacteria are generated by transforming bacteria with an expression plasmid (*pLux*) that contains the *luxCDABE* operon [5], [21]. Using this method, a mouse tumor model carrying grafted CT26 was injected with wild-type or GlmS^−^ bacteria carrying *^E.c^GlmS^+^pLux* via the tail vein. Expression of the *lux* gene was monitored using a cooled charge coupled device camera (Fig. 4A). Within 30 min of bacterial injection, bioluminescent signals were detected in the spleens and livers of the mice. At day 1, the signals from both types of bacteria had diminished in the liver but were detected exclusively in the tumor region. It should be noted that the signals from the GlmS^−^ mutant were significantly stronger than those from wild-type bacteria. Photon fluxes in tumor tissues were measured at the indicated days after the injection (Fig. 4B). The photon flux from GlmS^−^ mutant bacteria was 10- to 100-fold stronger than that from the wild-type *E. coli*. This was further confirmed by counting the number of CFU carrying the *^E.c^GlmS^+^pLux* (Fig. 4C). Tumor tissues were sampled on the indicated day, homogenized and spread on LB plates containing streptomycin and supplemented with GlcNAc for enumeration of the total number of bacteria, and on plates containing ampicillin and GlcNAc to assess the number of bacteria carrying the plasmid (Amp^R^). For both types of bacteria, the total number of bacteria increased from 10^7^ at day 1 to 5×10^9^ at day 5, and decreased gradually thereafter. However, the Amp^R^ wild-type bacteria decreased by approximately 50-fold by day 1, and approximately 1000-fold by day 5, and thereafter, while the number of GlmS^−^ mutant bacteria carrying the plasmid did not decrease. This suggested that the balanced-lethal system using *glmS* is effective within an animal system.

### Characterization of the Salmonella GlmS^+^ p vector

**Figure 5:**
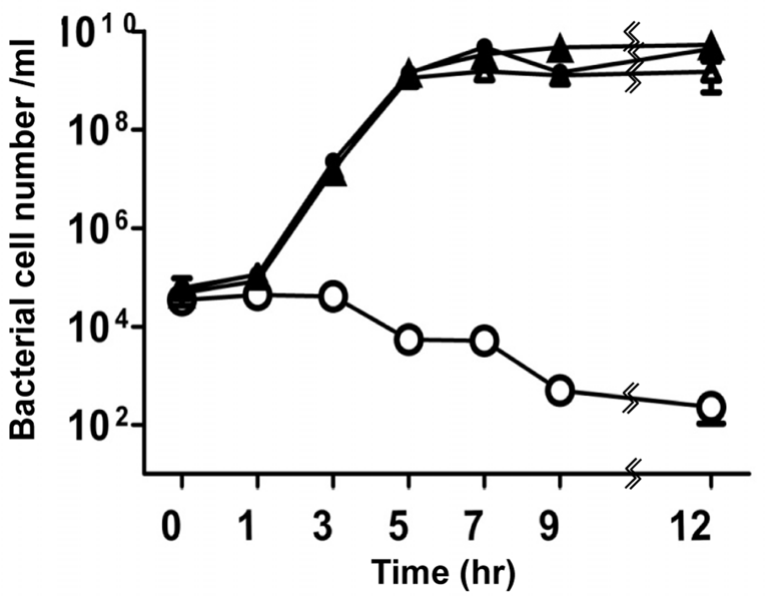
Phenotype of GlmS^−^ mutant *S. typhimurium*. (A) Growth of GlmS^−^ mutant *Salmonellae* under various media conditions. GlmS^−^ mutant *Salmonella* (SKS1001,open circles) and the GlmS^−^ mutant *Salmonella* carrying *^S.t^GlmS^+^p* (closed circles) grown overnight in LB supplemented with 0.2% GlcNAc were diluted 50-fold in minimal media or media supplemented with 0.2% GlcNAc (open triangles) and grown for 24 hrs. Wild-type parental *Salmonella* (SCH2005, closed triangles) were grown in the same way in minimal media. Samples were taken at the indicated times for CFU determination on supplemented LB plates.

In addition to *E. coli, Salmonella spp*. shown to be localized to transplanted tumors in animals has also been extensively developed to carry anti-tumoral cargo proteins [6]. Thus, we attempted to establish a balanced-lethal system in *Salmonella* using the *glmS* gene. First, the *glmS* gene on the chromosome of *S. typhimurium* was disrupted using the λ Red system [28]. A 1.8 Kb DNA fragment carrying *glmS* of *S. typhimurium* was placed under control of the *P_BAD_* promoter in the pBAD24 plasmid to construct the Salmonella *GlmS^+^* plasmid (*^S.t^GlmS^+^p*). The phenotype of GlmS^−^ mutant *Salmonella* was examined by culturing the mutant strain (SKS1001) in minimal media, as described previously for the *E. coli* mutant (Fig. 5). The assay revealed that the viability of the GlmS^−^ mutant was reduced from 5×10^4^ CFU to 5×10^2^ CFU over 12 hrs. In the presence of GlcNAc, however, the CFU of the mutant increased, similar to wild-type, from 5×10^4^ to 2×10^9^. In addition, GlmS^−^ mutant *Salmonella* carrying the *^s.t^*GlmS^+^ plasmid multiplied to a degree similar to that of the wild-type control in the absence of GlcNAc. Interestingly, *^E.c^GlmS^+^* failed to complement GlmS^−^ mutant *S. typhimurium*, even though the GlmS proteins of the two species are virtually identical: only 8 out of 609 amino acids differ (see NCBI sequence accession number: NP_418185 and YP_005240128 for *E. coli* and *S. typhimurium glmS* genes, respectively).

**Figure 6:**
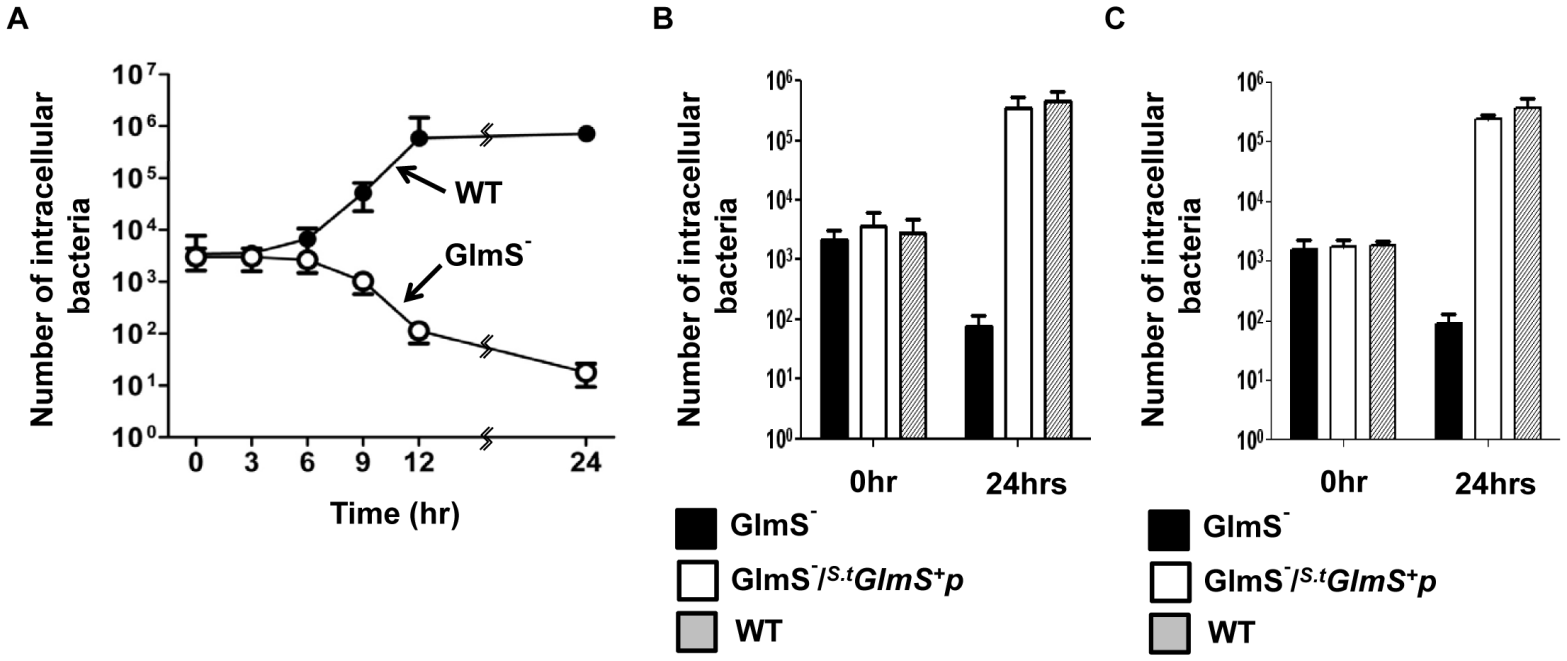
Intracellular growth of GlmS^−^ mutant *S. typhimurium*. (A) 5×10^5^ CFU of GlmS^−^ mutant (SKS1001) and wild-type (SCH2005) *Salmonellae* grown in LB supplemented with 0.2% GlcNAc was mixed with HeLa cells. Gentamycin-resistant intracellular *Salmonellae* were enumerated by determining the number of CFU at the indicated times. (B) The intracellular GlmS^−^ mutant bacteria, GlmS^−^ mutant bacteria carrying *^S.t^GlmS^+^p*, and wild-type bacteria were enumerated at 0 and 24 hrs in HeLa cells (B) and peritoneal macrophages (C).

*S. typhimurium* is capable of invading and replicating in animal cells [29]. Thus, the characteristics of GlmS^−^ mutant *S. typhimurium* was examined in cultured HeLa cells and peritoneal macrophages extracted from BALB/c mice. First, a time course assay was performed, in which intracellular bacteria were enumerated at 3 hr intervals. Wild-type and GlmS^−^ mutant Salmonellae grown in the presence of GlcNAc were mixed with HeLa cells and intracellular bacteria were counted in the presence of gentamycin (10 µg/ml)[30] (Fig. 6A and B). The number of bacteria that invaded HeLa cells were approximately equal for both wild-type and GlmS^−^ mutant bacteria (3∼4×10^3^ CFU at T = 0). The number of wild-type *Salmonellae* started to increase at 6 hrs post-infection and eventually reached 10^6^ CFU at 24 hrs post-infection. Conversely, the number of the GlmS^−^ mutant *Salmonellae* started to decline at 6 hrs post-infection and eventually reached less than 10 CFU at 24 hrs post-infection. This result again confirmed that the nutrients necessary for cell wall and membrane synthesis in GlmS^−^ mutant Salmonellae are not sufficiently present within animal cells. The decline in the number of intracellular GlmS^−^ mutant bacteria was most likely due to the effects of failed peptidoglycan synthesis, since onset of the decrease coincided with the time at which the number of wild-type bacteria began to increase (T = 6 hr). Subsequently, invasion and intracellular multiplication of GlmS^−^ mutant *Salmonellae* transformed with *^S.t^GlmS^+^p* was tested (Fig. 6B). Enumeration of intracellular bacteria at 0 and 24 hrs indicated that the complemented GlmS^−^ mutant bacteria invaded HeLa cells and multiplied intracellularly as effectively as wild-type bacteria. The same findings were obtained in peritoneal macrophages (Fig. 6C).

**Figure 7:**
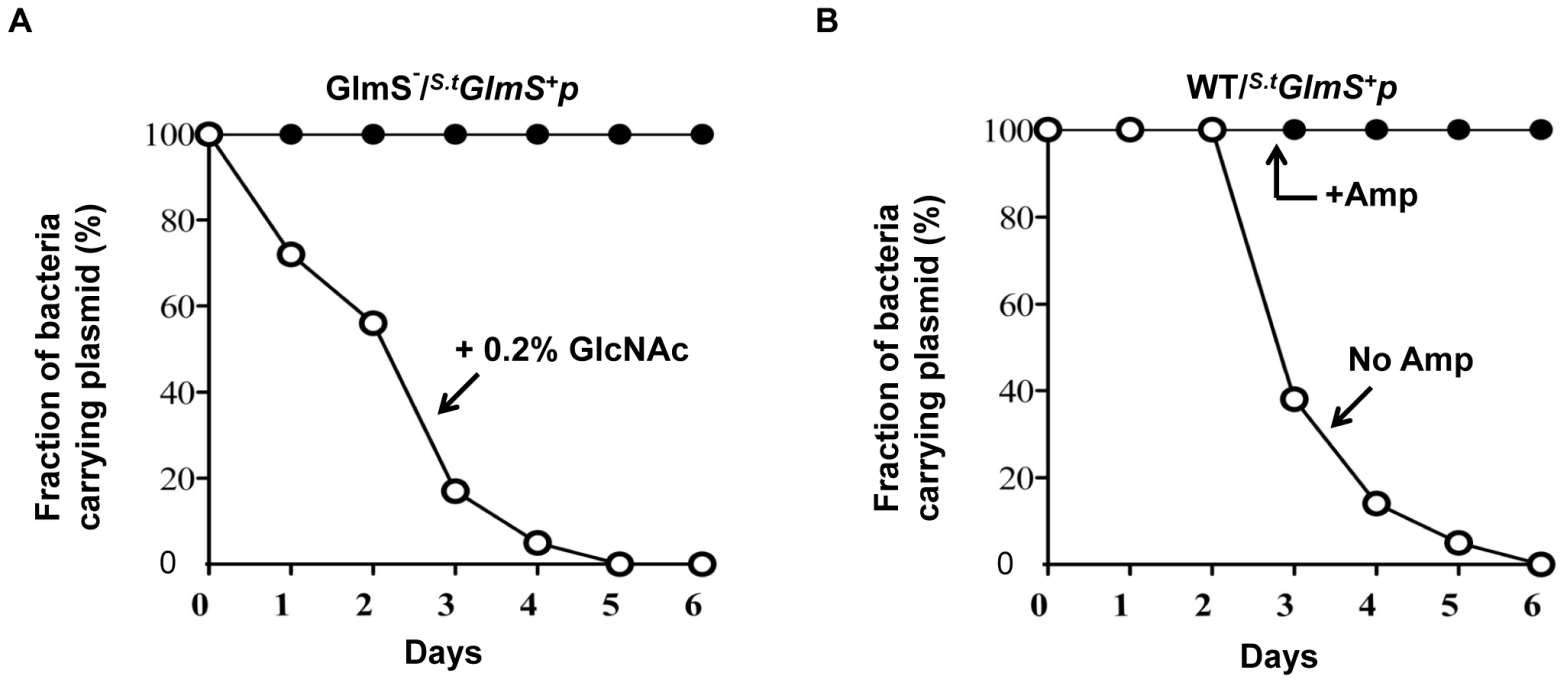
Plasmid maintenance in *S. typhimurium* using the *glmS* system *in vitro*. (A) GlmS^−^ mutant (SKS1001) salmonellae carrying *^s.t^GlmS^+^p* were subcultured (1/1000) in minimal media (closed circles) or media supplemented with 0.2% GlcNAc (open circles) every 12 hrs. The fraction of bacteria carrying *^s.t^GlmS^+^p* was determined on the indicated days by the plating method. (B) The same was done with wild-type *Salmonellae* (SCH2005) in the absence (open circles) or presence (closed circles) of ampicillin.

**Figure 8:**
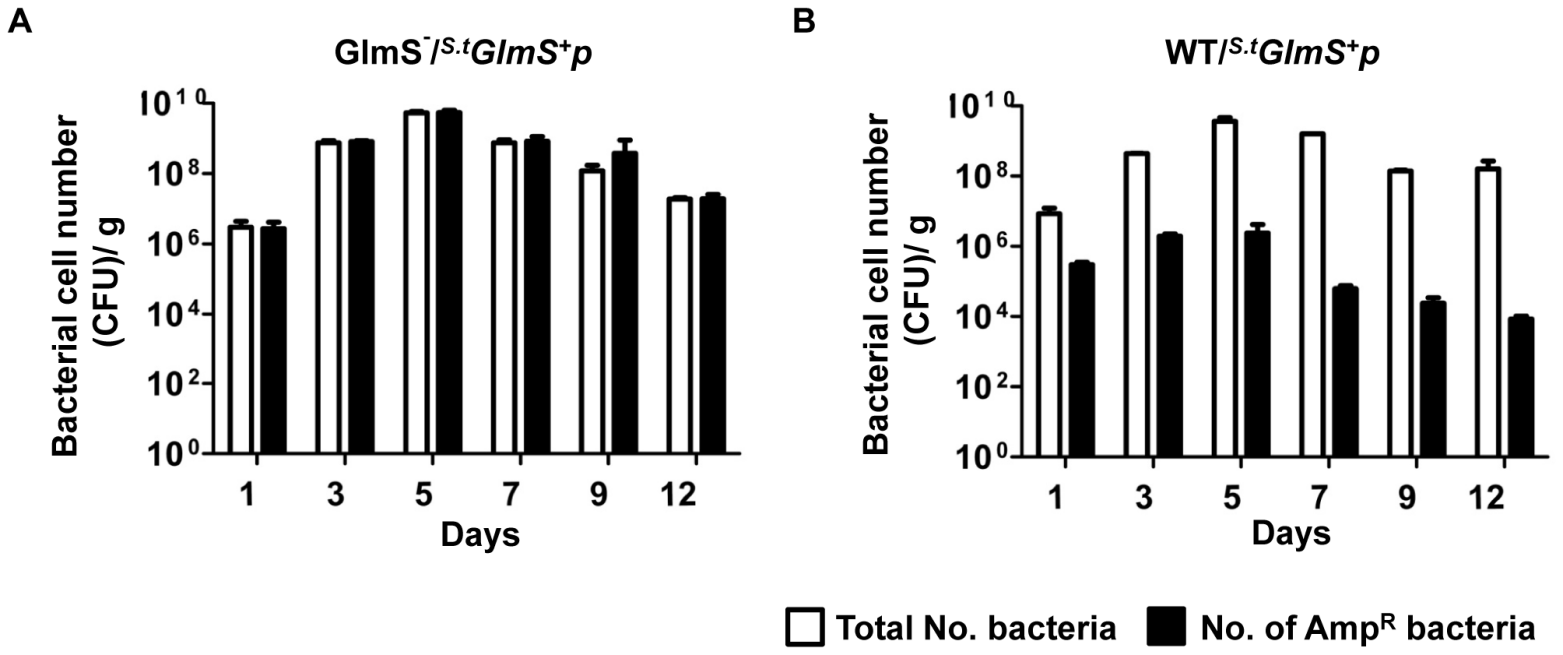
Maintenance of *^s.t^GlmS^+^p* in *S. typhimurium* proliferating in tumor tissue. (A) GlmS^−^ mutant (SKS1002, A) and parental *Salmonellae* (SHJ2037, B) carrying *^s.t^GlmS^+^p* were injected into CT26 tumor-bearing mice through the tail vein (3×10^7^ CFU). Tumor tissues were sampled on the indicated days, homogenized, and spread on GlcNAc-supplemented LB plates containing kanamycin and chloramphenicol for the enumeration of total number of bacteria and ampicillin for the determination of plasmid-carrying bacteria.

The fidelity of the balanced-lethal system using *glmS* was tested *in vitro* using wild-type and GlmS^−^ mutant *Salmonellae* carrying *^S.t^GlmS^+^p* (Fig. 7). In the wild-type background, the *^S.t^GlmS^+^p* were lost rapidly in the absence of antibiotics. In the GlmS^−^ mutant background, over 99% of *^S.t^GlmS^+^p* was lost at day 6 when cultured in media supplemented with GlcNAc, but strictly maintained when cultured in media lacking GlcNAc. Lastly, the *Salmonella glmS* balanced-lethal system was tested in an animal model with wild-type and GlmS^−^ mutant *Salmonellae* carrying *^S.t^GlmS^+^p*. Since *S. typhimurium* is highly virulent in rodents, an attenuated strain of *S. typhimurium* defective in ΔppGpp synthesis [2], [3] was used (ppGpp synthesized by *relA* and *spoT* is required for virulence of *S. Typhimurium* [31]). The ppGpp-null mutant (*relA::kan*, *spot::cat*) and the ppGpp-null mutant carrying the *glmS* mutation, which are both resistant to kanamycin and chloramphenicol, were transformed with ampicillin-resistant *^s.t^glmS^+^ p*. Mice carrying grafted CT26 (mouse colon cancer) cells were constructed as described previously. After 14 days, ΔppGpp or ΔppGpp/GlmS^−^ Salmonellae carrying *GlmS^+^pLux* (3×10^7^ CFU) were injected intravenously into each mouse via the tail vein. Tumor tissues were sampled on the indicated days, homogenized and spread on LB plates supplemented with GlcNAc containing kanamycin and chloramphenicol and/or ampicillin. Both the total number of bacteria (Kan^R^ Cat^R^) and the number of bacteria carrying the plasmid (Amp^R^) were counted (Fig. 8). The total number of both types of bacteria increased from 10^7^ at day 1 to 5×10^9^ at day 5 and decreased gradually thereafter. However, the number of Amp^R^ parental bacteria was 50-fold less at day 1, 5000-fold less at day 5 and more than 10,000-fold less at day 12. Conversely, the number of Amp^R^ GlmS^−^ mutant bacteria was the same as the total number of bacteria. This was further verified using two other tumor models: BALB/c mice carrying 4T-1 (mouse breast cancer) and nude mice carrying ASPC-1 (human pancreatic cancer). The numbers of bacteria carrying the plasmid were counted at day 7 in the homogenized tumor tissue (Table 2). Similarly as with CT26-bearing mice, virtually all GlmS^−^ mutant *Salmonella* carried the plasmid, but only ∼0.1% wild type *Salmonella* carried the plasmid irrespective of types of tumor models. These data demonstrated that the *Salmonella glmS* balanced-lethal host-vector system ensured maintenance of the plasmid in the absence of a selective determinant in animals.

**Table 2:** Maintenance of *^S.t^GlmS^+^p* in *S. typhimurium* proliferating in tumor tissues.

Designation	Origin	GlmS		WT	
		Km^R^, Cm^R^	Amp^R^/Km^R^, Cm^R^	Km^R^, Cm^R^	Amp^R^/Km^R^, Cm^R^
ASPC-1	*Pancreatic adenocarcinoma*	1.9×10^8^	2.4×10^8^	3.0×10^8^	3.0×10^5^
		±4.4	±4.81	±3.29	±2.69
4T-1	*Murine mammary carcinoma*	6.6×10^8^	4.7×10^8^	4.4×10^8^	1.4×10^5^
		±1.28	±2.16	±2.82	±4.4

*SMR2130* (GlmS^−^) and *SKS1002* (WT) strains carrying *^S.t^GlmS^+^pLux* (3×10^7^ CFU), were injected intravenously into mouse bearing 4T-1 (mouse breast cancer) or ASPC-1 (human pancreatic cancer). Tumor tissue were sampled at 7 days after the injection, homogenized, spread on LB plates containing kanamycin and chloramphenicol and/or amphicilin, and enumerated total number of bacteria (Km^R^ Cm^R^) and those carrying plasmid(Amp^R^).

## Discussion

**Figure 9:**
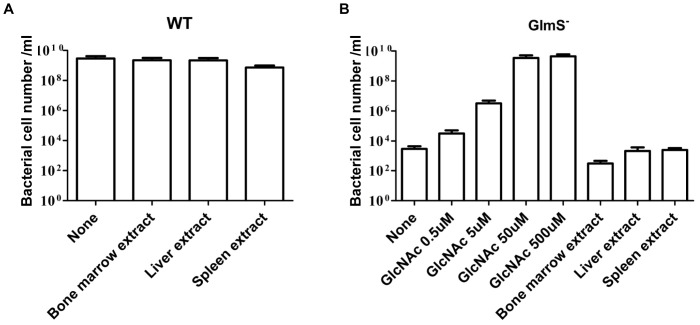
Growth of WT (CH1018, A) and GlmS^−^ (CKS1001, B) mutant *E. coli* in the minimal media with supplementations. ∼5×10^5^ CFU/ml of bacteria were inoculated in the M9 media containing indicated supplementations, grown for 24 hrs at 37°C, and total numbers of bacteria were enumerated on minimal media plates containing 500 mM GlcNAc. Final concentrations of each organ extracts were 0.2 mg/ml.

The system based on the *asd* gene is the most acclaimed balanced-lethal host system [7]. Here, we have demonstrated that, as for the Asd^−^ mutant, animal tissues lack the nutrients required for survival of GlmS^−^ mutant bacteria (Fig. 3). Since the level of DAP is insufficient in mammalian tissue, the balanced-lethal system of *asd* system coerces multiplying Asd^−^ mutant *Salmonella* within the animal to carry the recombinant Asd^+^ plasmid [8] [13]. In this study, we presented multiple lines of evidence demonstrating that GlmS^−^ mutant *E. coli* and *S. typhimurium* undergo lysis unless GlcNAc is supplied exogenously and/or the bacteria are complemented by an *^E.c^GlmS^+^p* or *^S.t^GlmS^+^p* vector, respectively. For the successful application of a balanced-lethal host system based on *glmS*, would be insufficient supply of those intermediates fot synthesis of GlcNAc in animal tissues. In mammals, GlcNAc is a component of glycoproteins, proteoglycans, glycosaminoglycans (GAGs) and other connective tissue building blocks [32]. Despite being a building block of biomacromolecules, GlcNAc seldom exists in free form [33]. We determined the growth of GlmS^−^ mutant *E. coli* in the presence of bone marrow, spleen, and liver extract, found the supplementations did not support the growth of the mutants (Fig. 9). GlmS^−^ mutant *E. coli* required ∼50 mM GlcNAc for normal growth. Thus, the necessary intermediates for the synthesis of GlcNAc are present at insufficient levels in these animal tissues to sustain the proliferation of GlmS^−^ mutant bacteria. This is consistent with the recent determination, median endogenous glucosamine concentrations in plasma and synovial fluid in human were 0.29 µM and 0.21 µM, respectively [34].

Lytic cell death of GlmS^−^ in the absence of GlcNAc supply was demonstrated to be due to leakage of cellular contents (Figs. 2 and 4). It should be noted that bacteria apparently fail to detect the absence of the building blocks necessary for membrane synthesis and continue to expand until they undergo lysis. This is in contrast to the situation that occurs in the absence of sufficient supply of amino acids or nucleotides, under which conditions bacteria cease proliferation through the accumulation of ΔppGpp [27]. The balanced-lethal host-vector system takes advantage of this phenomenon to ensure that the bacteria maintain the plasmid under *in vivo* conditions in which there is no selective pressure.

The observation that up to 99.99% of wild-type bacteria abandoned the GlmS^+^ plasmid within 3–5 days after *i.v*. injection was remarkable (Figs. 4 and 8). It indicates that maintenance of a plasmid that is not needed for survival imposes a great stress on bacteria, especially *in vivo*, where bacteria must struggle to ensure they acquire the nutrients necessary for survival while escaping the immunological assault of the host animal. This observation underscored the capacity of a balanced-host lethal system to maintain plasmids carrying genes for therapeutic proteins. Bioluminescent signals from the GlmS^−^ mutant were up to several hundred-fold stronger than those from wild-type bacteria (Fig. 4). This suggests that bacterial therapies utilizing *S. typhimurium* carrying a plasmid containing an anti-tumoral protein gene [4], [6] would be significantly more effective in a *glmS-*based balanced-lethal system.

While *in vivo* imaging of bioluminescent bacteria is a powerful tool that allows us to visualize the process of bacterial-tumor targeting, quantify bacterial growth noninvasively in target tissues, and monitor bacterial migration in real time, [5] it requires direct correlation between the photon flux and the number of bacteria. The plasmid (*pLux*) that contains the *luxCDABE* operon from *Photobacterium leiognathi* employed in this study is appropriate for this since it does not require an exogenous source of substrate to produce bioluminescence [5], [35]. This allows direct measurement of photon flux from bacteria in deep tissues without the inconvenience of light scattering and attenuation through body tissues due to the lack of an excitation source. In the absence of selection pressure, however, the loss of plasmid carrying *lux* operon would be an obstacle. The *pLux* expression vector loaded with the *GlmS^+^p*/ΔglmS balanced-lethal system offers a solution that allows for the direct quantification of bacteria within living animals by determination of bioluminescence via IVIS imaging.
